# Corn starch reactive blending with latex from natural rubber using Na^+^ ions augmented carboxymethyl cellulose as a crosslinking agent

**DOI:** 10.1038/s41598-021-98807-x

**Published:** 2021-09-28

**Authors:** Noppol Leksawasdi, Thanongsak Chaiyaso, Pornchai Rachtanapun, Sarinthip Thanakkasaranee, Pensak Jantrawut, Warintorn Ruksiriwanich, Phisit Seesuriyachan, Yuthana Phimolsiripol, Charin Techapun, Sarana Rose Sommano, Toshiaki Ougizawa, Kittisak Jantanasakulwong

**Affiliations:** 1 School of Agro-Industry, Faculty of Agro-Industry, Chiang Mai University, Mae Hia, Muang, Chiang Mai Thailand; 2 Cluster of Agro Bio-Circular-Green Industry, Faculty of Agro-Industry, Chiang Mai University, Mae Hia, Muang, Chiang Mai Thailand; 3 Center of Excellence in Materials Science and Technology, Faculty of Science, Chiang Mai University, Mae Hia, Muang, Chiang Mai Thailand; 4 Department of Pharmaceutical Sciences, Faculty of Pharmacy, Chiang Mai University, Mae Hia, Muang, Chiang Mai Thailand; 5 Plant Bioactive Compound Laboratory (BAC), Department of Plant and Soil Sciences, Faculty of Agriculture, Chiang Mai University, Mae Hia, Muang, Chiang Mai Thailand; 6 Department of Chemistry and Materials Science, Tokyo Institute of Technology, Meguro-ku, Tokyo, Japan

**Keywords:** Chemistry, Materials science

## Abstract

A mixture of corn starch and glycerol plasticizer (CSG) was blended with latex natural rubber (LNR) and carboxymethyl cellulose (CMC). The addition of 10 phr of CMC improved the Young’s modulus (6.7 MPa), tensile strength (8 MPa), and elongation at break (80%) of the CSG/LNR blend. The morphology of the CSG/LNR/CMC blends showed a uniform distribution of LNR particles (1–3 µm) in the CSG matrix. The addition of CMC enhanced the swelling ability and water droplet contact angle of the blends owing to the swelling properties, interfacial crosslinking, and amphiphilic structure of CMC. Fourier transform infrared spectroscopy confirmed the reaction between the C=C bond of LNR and the carboxyl groups (–COO^−^) of CMC, in which the Na^+^ ions in CMC acted as a catalyst. Notably, the mechanical properties of the CSG/LNR/CMC blend were improved owing to the miscibility of CSG/CMC and the CMC/LNR interfacial reaction. The CSG/LNR/CMC biodegradable polymer with high mechanical properties and interfacial tension can be used for packaging, agriculture, and medical applications.

## Introduction

Biodegradable polymers have attracted considerable attention because they are environmentally friendly and readily degradable. They can be particularly competitive in certain sectors of the plastics market, such as food packaging^1,2^ at both the industrial and street food levels, where hygiene must be practiced to prevent packaging contamination^3^. Petroleum-based polymers generated from petroleum resources subject to depletion have detrimental effects on the environment^4,5^. Therefore, biodegradable polymers are being widely investigated to replace petroleum polymers, with examples including polylactic acid (PLA)^6,7^, polybutylene succinate (PBS)^8,9^, thermoplastic starch (TPS)^10^, chitosan^11,12^, pectin^13–15^, polysaccharides^16,17^, keratin^18^, and fibroin^19^.

Starch is a natural polymer possessing desirable traits such as complete biodegradability, low cost, and renewability. Starch is produced from plants that are widely consumed by humans. Starch is a semi-crystalline biopolymer whose structure contains starch granules with different amylose/amylopectin ratios, depending on the starch resources and gelling properties of water and heat^20^. Starch processing involves chemical reactions, including melting, gelatinization, and water diffusion^21^. Some common resources for the production of starch have been investigated, such as cassava^22^, mung beans^23^, and corn^24^, along with the development of special enzymes that can degrade starch under harsh conditions^25^. Corn starch (CS) is the world’s largest starch resource^26^ and is widely used in food processing^27^ and food packaging^28^.

Natural rubber (NR) is composed of *cis*-1-s-polyisoprene, which is extracted from the para rubber tree (*Hevea brasiliensis*) to obtain latex^29^. This latex from NR (LNR) has a relatively high water content and up to 60% rubber content. LNR contains rubber particles, lipids, carbohydrates, and proteins in aqueous solution. Skim latex is purified using a creaming agent and saponification^30^. Ammonia is a common chemical used to prevent coagulation and bacterial growth in LNR^31^. Because of its excellent properties, LNR is used extensively to manufacture several products (e.g., gloves, balloons, rubber boots, and condoms). The mechanical properties of rubber are improved by crosslinking in the rubber phase^32^. LNR is also the main natural polymer resource of the global agricultural economy^33^; however, NR prices have dropped because of economic conditions^34^. There have been some studies on NR blending utilizing starch^35^. The mechanical properties of NR blends can be improved by crosslinking certain functional groups in rubber using various methods^36^.

Carboxymethyl cellulose (CMC) is a cellulose derivative obtained from alkali cellulose and sodium salt reactions^37^. The main applications of CMC include food, paper, printing, medicine, and packaging^38^. CMC contains carboxylic groups with Na^+^ ions^39^ and exhibits high viscosity and nontoxic properties. CMC has been prepared using various source materials such as asparagus^40^, palm bunches^41^, bacterial cellulose^42,43^, and chitosan^44^. CMC can also act as a compatibilizer to improve the properties of starch^45^. The mechanical properties of CMC/natural rubber blends with polyaniline^46^ and cellulose^47^ have been previously reported. Azura et al. presented the interaction between nano-starch filler and LNR, which could improve the mechanical properties of the blends^48^. However, few studies have investigated the improvement in the mechanical properties of starch blended with NR undergoing a reaction with CMC.

The aim of this study was therefore to develop biopolymer films with good mechanical properties using reactive blending of CS and glycerol (CSG), LNR, and CMC. CS was selected as the main matrix for the blend because of its biodegradability, high purity, chemical modification abilities, low cost, and abundance. Glycerol was used as a plasticizer to improve the flexibility and processing ability of the CS. LNR was blended with CSG to enhance the toughness and flexibility as an elastic phase of the blends. CMC was used as a crosslinking agent. It was suggested that the high compatibility of CS/CMC and the reaction between CMC and LNR would improve the mechanical properties of the blends. A tough, transparent, water-resistant biodegradable material with high tensile properties was developed. The effects of CMC addition and the presence of Na^+^ ions in the CMC were investigated. The tensile properties, morphology, water resistance, and reaction mechanisms were also evaluated, resulting in a high tensile strength biomaterial made from a starch/natural rubber blend for packaging, agriculture, and medical applications.

## Results and discussions

### Reaction mechanism

**Figure 1:**
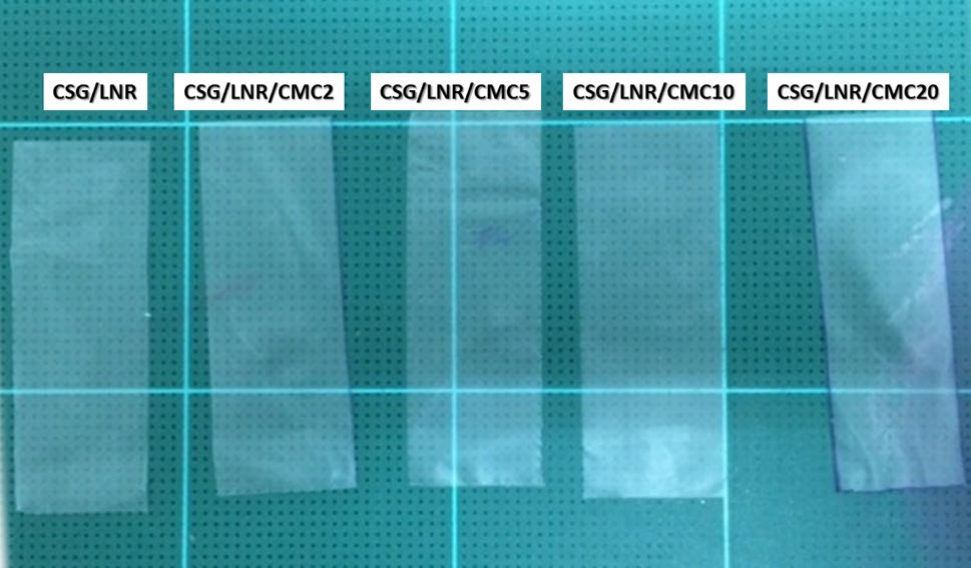
Image of CSG/LNR films blending with CMC 0, 2, 5, 10, 20 phr.

**Figure 2:**
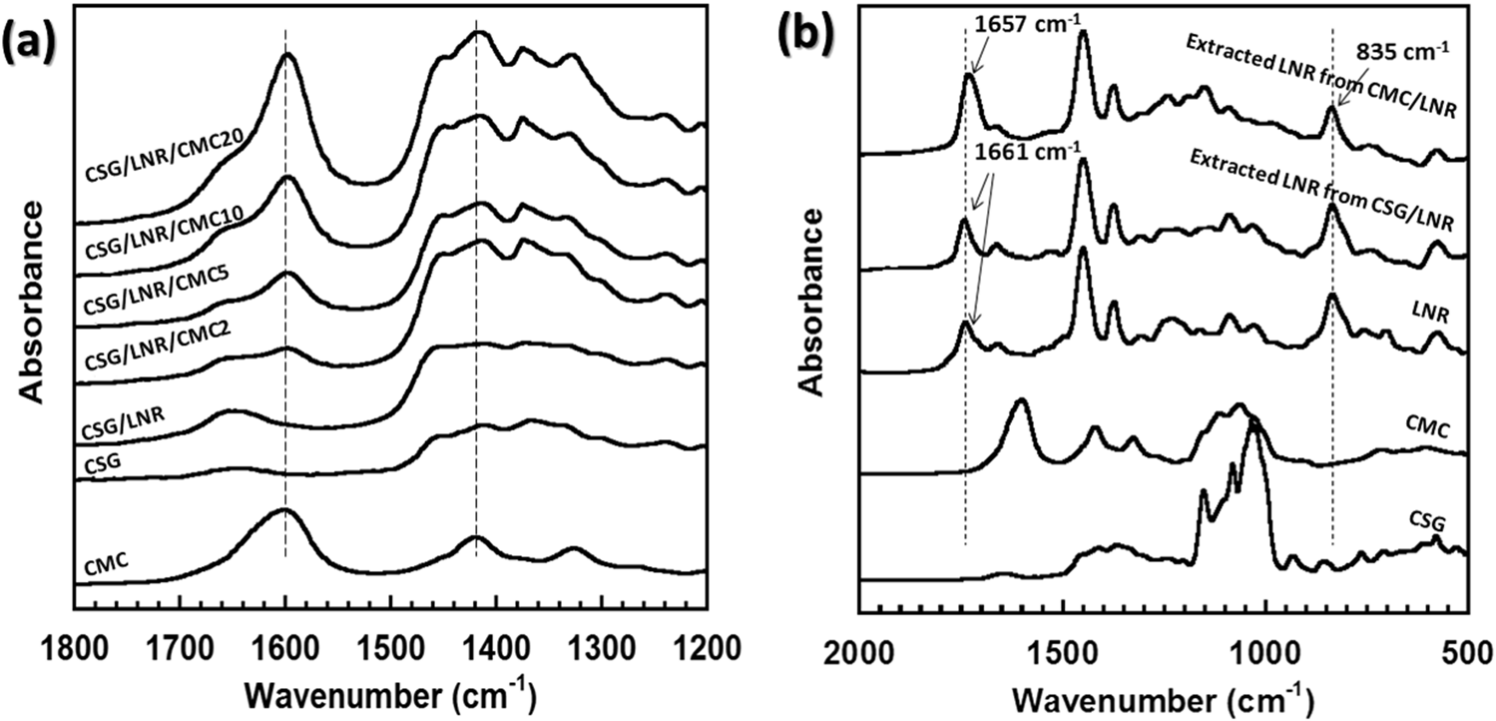
FTIR spectra of (**a**) CMC, CSG, CSG/LNR blend with CMC 0–20 phr at 1200–1800 cm^−1^, and (**b**) CSG, CMC, LNR, and the extracted LNR from CSG/LNR and CMC/LNR. at 500–2000 cm^−1^.

**Figure 3:**
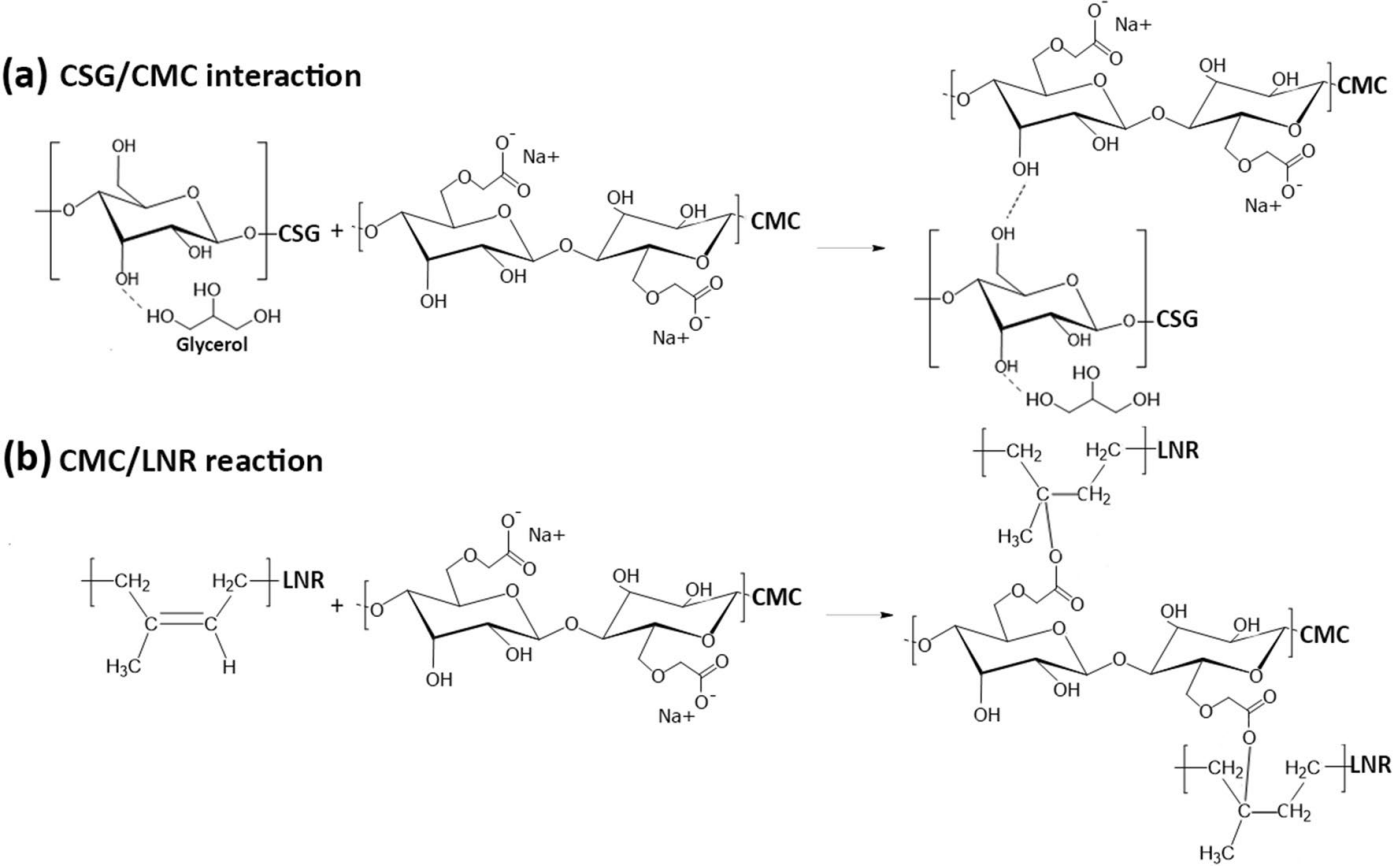
Suggested reaction of (**a**) interaction between CSG and CMC and (**b**) reaction between CMC and LNR.

CS with glycerol plasticizer (CSG) was blended with CMC and LNR through solution mixing at 80 °C for 1 h. The mixed solutions were applied to films and dried at 60 °C for 24 h. Images of the CSG/LNR blend with CMC 0–20 phr samples are shown in Fig. 1. The reaction mechanisms of CSG, CMC, and LNR were investigated using FTIR spectroscopy. Figure 2a shows the FTIR spectra of CMC, CSG, and CSG/LNR blends with 0–20 phr CMC. The FTIR spectra of LNR (*cis*-1,4 polyisoprene) exhibited C‒H stretching (2960, 2927, and 2852 cm^−1^), C=C stretching (1661 cm^−1^), C‒H deformation of stretching ‒CH_2_‒ (1448 cm^−1^), C‒H deformation of ‒CH_3_ (1376 cm^−1^), and C=C‒H (835 cm^−1^)^49,50^. The CSG spectra exhibited peaks at 1643 (‒OH bending), 1016, and 929 cm^−1^ (‒CO stretching)^51,52^. The CMC spectra exhibited peaks at 3040 (‒OH stretching), 2897 (‒CH stretching), 1602 (COO^−^), and 1427 cm^−1^ (COO^−^Na^+^)^53,54^. The spectra of the CSG/LNR blend exhibited a combination of the individual CSG and LNR spectra. The CSG/LNR/CMC blend presented an increase in peak intensities at 1602 (COO^−^) and 1427 cm^−1^ (COO^−^Na^+^) of CMC. To study the reaction mechanism of the blend, the LNR phase was extracted from the CSG/LNR and CSG/LNR/CMC blends. The spectra of CSG, CMC, LNR, and the extracted LNR are shown in Fig. 2b. The CH_2_‒ peak at 1448 cm^−1^ of the LNR was used to normalize the extracted LNR samples. The extracted LNR from the CSG/LNR exhibited spectra similar to those of pure LNR, with peaks at 1661 (C=C stretching) and 835 cm^−1^ (C=C‒H). Furthermore, in the LNR extracted from the CSG/LNR/CMC blend, the peak at 1661 cm^−1^ (C=C) shifted to 1657 cm^−1^ and increased in intensity. This indicates a new ‒C‒O peak due to the reaction between CMC and LNR. The intensity of the peak at 835 cm^−1^ (C=C‒H) decreased compared to that of pure LNR owing to the reduction of the C=C‒H structure in the LNR chain. The Na^+^ ion in CMC is in the form of a Lewis acid catalyst^55^. Crosslinking at the C=C structure of NR was accelerated by the Lewis acid catalyst, as reported previously^56^. Supanakorn et al.^47^ confirmed the interaction between CMC and LNR, which improved the mechanical properties of the LNR/cellulose/CMC blend. It has also been reported that Na^+^ ions inside the CMC accelerate the reaction through its COO^−^ groups^45^. It was confirmed that the C=C of the LNR structure reacted with the COO^−^ of CMC as the Na^+^ ion in CMC acted as a catalyst. The suggested reaction mechanism is illustrated in Fig. 3. CSG showed high compatibility with CMC owing to their structural similarity and interaction between the ‒OH groups (Fig. 3a), whereas a reaction occurred between the C=C of LNR and COO^−^ of CMC (Fig. 3b).

### Mechanical properties

**Figure 4:**
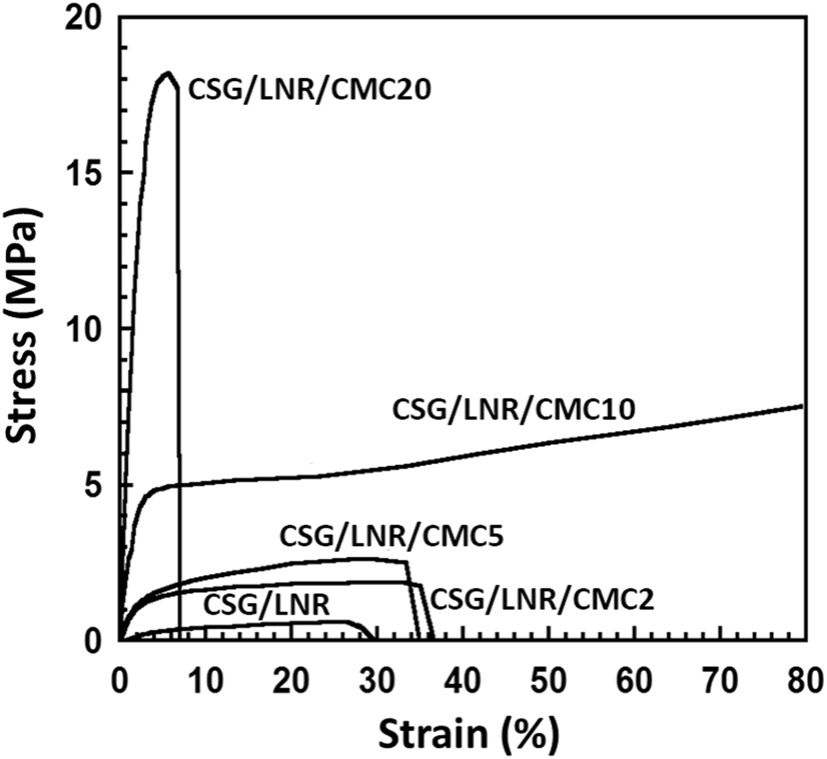
Tensile stress–strain curves of the CSG/LNR blends with CMC of 0, 2, 5, 10, and 20 phr (n = 5).

Figure 4 shows the stress–strain curves of the CSG/LNR blends with 0–20 phr CMC. The Young’s modulus was calculated from the slope at the early stage of the stress–strain curve. The CSG/LNR blend showed a low Young’s modulus of 0.3 MPa, a maximum tensile strength of 0.5 MPa, and an elongation at break of 30% (Table 1). The addition of CMC resulted in an increase in Young’s modulus and maximum tensile strength. The CSG/LNR/CMC10 blend exhibited a Young’s modulus of 6.7 MPa, a maximum tensile strength of 8 MPa, and an elongation at break of 80%, with all values higher than those for the blends where CMC was added at 0, 2, and 5 phr. The CSG/LNR/CMC20 blend exhibited the highest Young’s modulus (18.2 MPa) and maximum tensile strength (18 MPa) and the lowest elongation at break because of the high interfacial crosslinking density, reaction mechanism, and high mechanical properties of CMC. The tensile strength, elongation at break, and toughness of the CSG/LNR blend were improved by adding CMC, particularly for the CSG/LNR/CMC10 sample compared to the CSG/LNR blend. The toughness of the sample is related to the area under the stress–strain curve^57^. The Young’s modulus of starch increased with the CMC content^58^, and a high interfacial reaction improved the mechanical properties of the polymer blends, which has been reported previously^59^. An improvement in the mechanical properties of natural rubber by the addition of CMC has also been reported^46,47^. CMC was found to improve the interfacial adhesion between CSG and LNR through CMC crosslinking as a compatibilizer. CMC is compatible with starch, carboxylic groups, and sodium ions in its structure^45^. These induced the formation of crosslinking between CSG and LNR through CMC. CMC acted as a physical crosslinking point to connect the structures of starch and rubber together, which provided the combined properties of CSG, CMC, and LNR. In the CSG/LNR/CMC sample, CMC 10% was suitable for connecting CSG (hard phase) and LNR (elastic phase), providing high elongation at breaking. In the CSG/LNR/CMC20 sample, high content, mechanical properties, and physical crosslinking of CMC increased the tensile strength and brittleness of the blend. The improvement in the tensile properties was attributed to the compatibility of CSG/CMC and the occurrence of the interfacial crosslinking density of CMC/LNR through a reaction mechanism between the C=C of LNR and COO^−^ of CMC (Fig. 3b).

**Table 1:** Young's modulus, maximum tensile strength, and elongation at break of CSG/LNR blends with 0–20 phr CMC.

Sample	Young's modulus (MPa)	Maximum tensile strength (MPa)	Elongation at break (%)
CSG/LNR	0.3 ± 0.08^a^	0.5 ± 0.08^a^	30.1 ± 2.40^b^
CSG/LNR/CMC2	2.4 ± 0.07^b^	1.7 ± 0.12^b^	35.4 ± 2.89^c^
CSG/LNR/CMC5	2.8 ± 0.08^c^	2.9 ± 0.21^c^	33.3 ± 3.21^c^
CSG/LNR/CMC10	6.7 ± 0.08^d^	8.0 ± 0.43^d^	79.9 ± 4.35^d^
CSG/LNR/CMC20	18.2 ± 0.08^e^	18.0 ± 2.1^e^	7.80 ± 0.51^a^

Means with different lowercase superscript letters in the same column are significantly different (P < 0.05).

### Morphology

**Figure 5:**
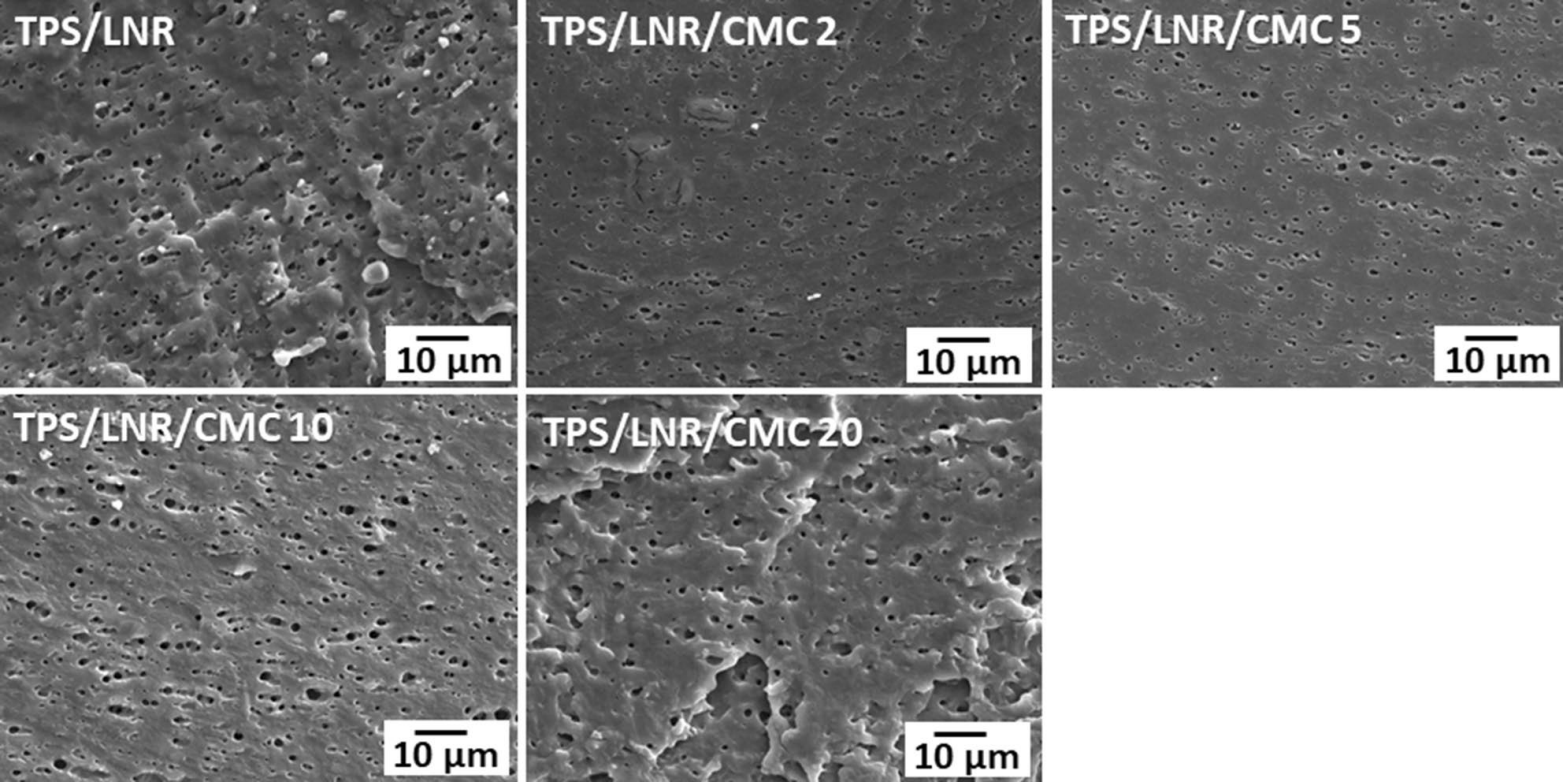
SEM images of CSG/LNR blends with CMC 0, 2, 5, 10, and 20 phr.

The morphologies of the samples were observed using SEM. The samples were broken in liquid nitrogen, and then the LNR phase on the fracture surfaces was extracted by immersion tin toluene at 60 °C for 1 h. Figure 5 shows the fracture surface images of the CSG/LNR and CSG/LNR/CMC blends with 2, 5, 10, and 20 phr of CMC. The CSG/LNR blend exhibited voids representing the LNR particles extracted using toluene because NR dissolves in toluene^60^. ImageJ software was used to evaluate the rubber particle sizes. The LNR particle sizes in the CSG/LNR blend were 1–3 µm. The addition of CMC at 2, 5, 10, and 20 phr resulted in the dispersion of the LNR rubber particles (1–3 µm) in the CSG matrix. The LNR formed small rubber particles in the CSG matrix, while the addition of CMC did not reduce the particle size of the LNR. The improvement in the tensile properties was probably due to the high tensile properties of CMC, interfacial crosslinking density of CSG/LNR, and crosslinking density inside the LNR phase.

### Solubility and swelling

**Figure 6:**
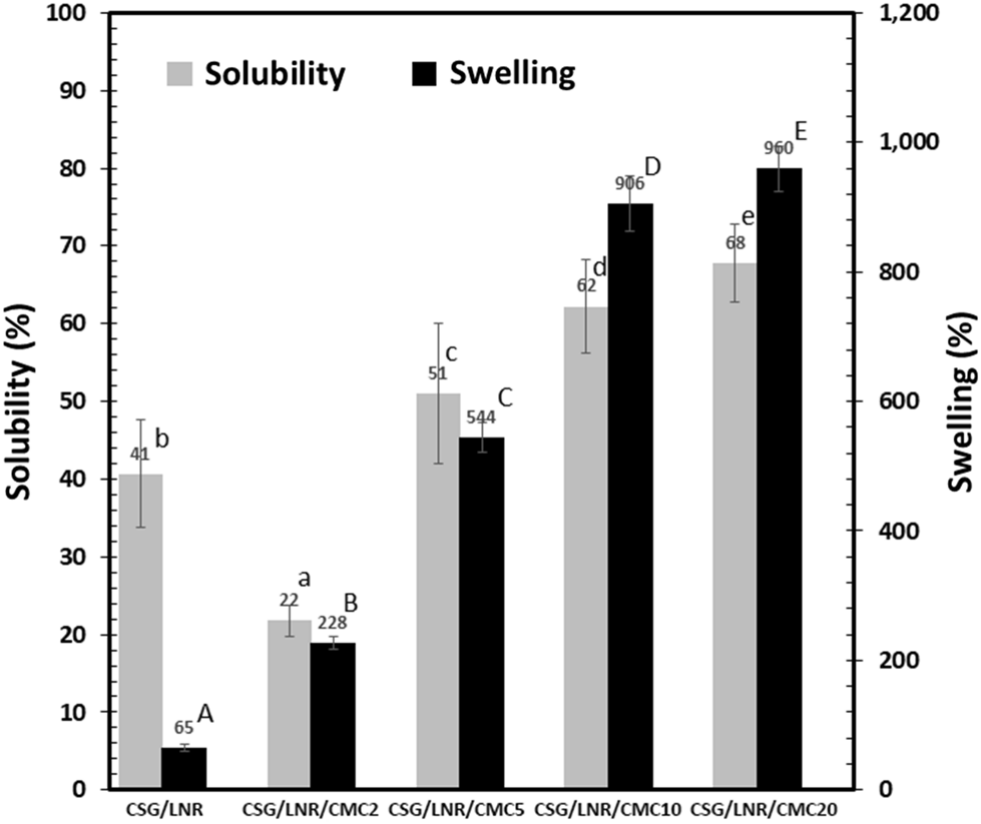
Solubility and swelling of the CSG/LNR/CMC blends with 0, 2, 5, 10, and 20 phr of CMC (n = 5). Means with different lowercase letters of solubility and uppercase letters of solubility are significantly different (P < 0.05).

The solubility and swelling of the samples were measured by immersing the samples in distilled water at 25 °C for 24 h. The CSG film was prepared by the controlled mixing of starch with glycerol (70/30%w/w). The solubility and swelling degree of the CSG/LNR film were 41% and 65%, respectively (Fig. 6). Solubility was calculated from the weight loss of the samples in water. The solubility of CSG/LNR/CMC2 decreased to 22% owing to the formation of interfacial crosslinking density between CMC and LNR. The elevated CMC content increased the solubility of the CSG/CMC/LNR blends because of the high soluble material content in the blends. The CSG/LNR showed low swelling owing to the low crosslinking density between the CSG and LNR phases. The degree of swelling increased with the CMC content due to the increase in the interfacial crosslink density of CSG/LNR through the CMC reaction mechanism (Fig. 2), and the crosslinking inside the CMC phase through Na^+^ ions. The CMC structure contains Na^+^ ions from its synthesis process, which form physical crosslinking with the COOH groups of CMC^61^. CS is a hydrophilic material^62^, whereas CMC forms a gel in water^37,41^. The increase in the swelling degree was evidence of the hydrophilic properties of CS and CMC^62,63^, the swelling ability of CMC^64^, and the interfacial crosslinking density of CSG/LNR through the CMC reaction mechanism.

### Contact angle

**Figure 7:**
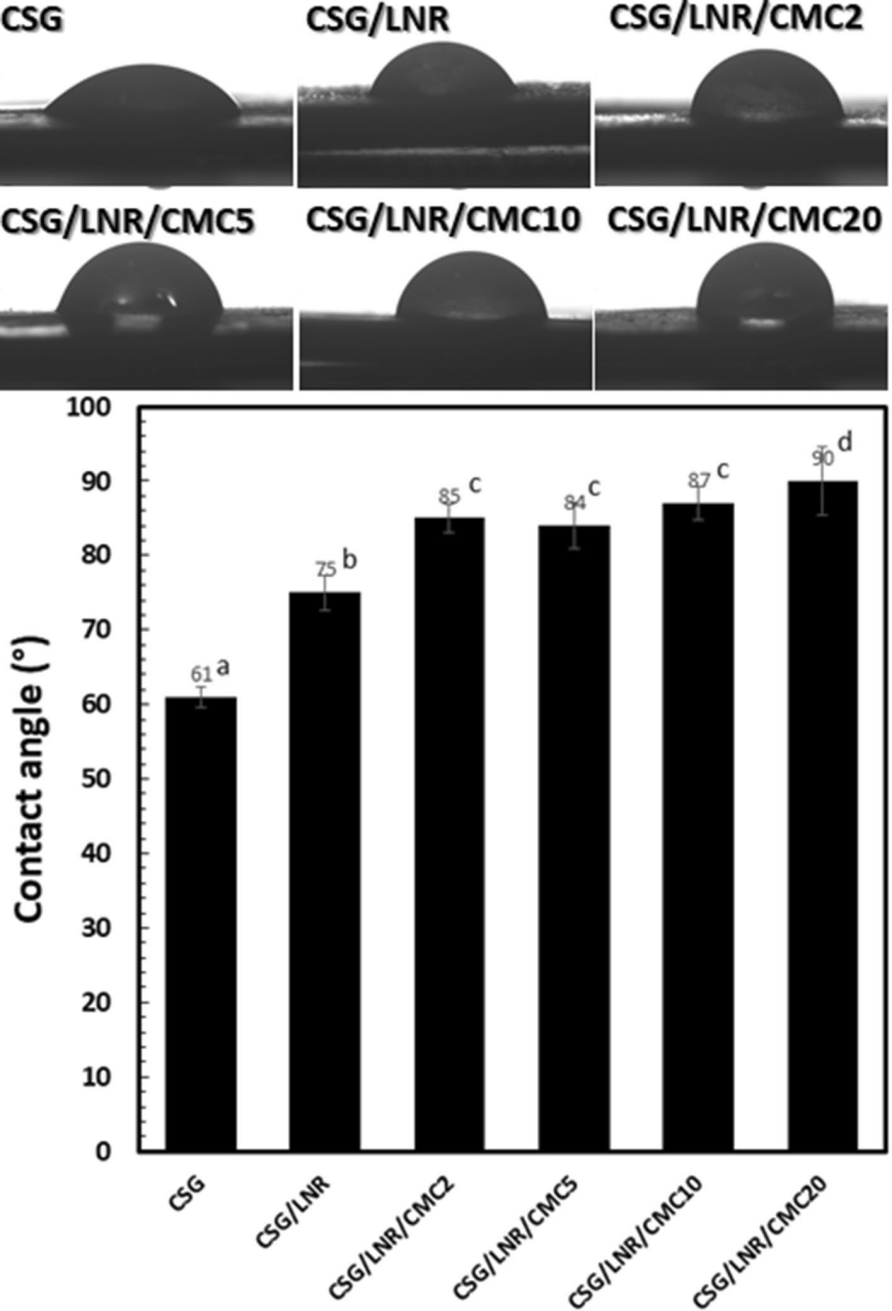
Contact angles of CSG and the CSG/LNR blends with 0, 2, 5, 10, and 20 phr of CMC at 3 min (n = 5). Means with different lowercase superscript letters are significantly different (P < 0.05).

The water droplet contact angle is related to the hydrophilicity, crosslinking, and surface tension of the materials. Figure 7 shows the contact angle of CSG and the CSG/LNR blends with 0–20 phr of CMC at 3 min. CSG exhibited a low contact angle of 61°. The contact angle of the CSG/LNR blends increased with increasing CMC content, especially at 20 phr. CS is a polar material, whereas amphiphilic CMC combines polar and nonpolar structures^63^. The increase in the contact angle of the CSG/LNR blend was probably caused by small hydrophobic rubber particles that were finely dispersed in the CSG matrix. The increase in the contact angle of CSG/LNR/CMC2 may be due to the combination of the interfacial crosslinking density between LNR and CMC, hydrophobicity of LNR, and the non-polar portion of CMC. Particles of hydrophobic LNR with a crosslinked phase through CMC increased the interfacial tension and repelled the water droplet from the surface. The addition of 5–20 phr of CMC increased the contact angle to 85–90°, owing to the enhanced non-polar portion of CMC and interfacial crosslinking density.

## Conclusions

A new biopolymer film with improved mechanical properties and interfacial tension was successfully developed by blending CSG with CMC and LNR. The incorporation of CMC into the CSG/LNR blend enhanced the tensile properties of the blend because of the improvement in the interfacial reaction, miscibility of CSG/CMC, LNR crosslink, and mechanical properties of CMC. The interfacial crosslinking density of CMC/LNR improved the solubility of the CSG/CMC/LNR blend. The swelling properties were enhanced with CMC content owing to the gel formation of CMC. The contact angle increased with the CMC content owing to the hydrophobic nature of LNR, interfacial crosslinking density of CMC/LNR, and non-polar structural portion of the amphiphilic CMC. The FTIR results confirmed the reaction between the COO^−^ groups of CMC and the C=C groups of LNR in the presence of Na^+^ ions acting as Lewis acid catalysts. This reaction mechanism and the compatibility of CSG/CMC improved the mechanical properties and interfacial tension of the CSG/CMC/LNR blend. The CSG/CMC/LNR blend, with its excellent properties, can be used in packaging, agriculture, and medical applications.

## Methods

### Materials

CS (Super-Find brand with MW of 2.54 × 10^8^ g/mol) was procured from R&B Food Supply Public Co. Ltd., Bangkok, Thailand. Glycerol (99%) was procured from Yok Inter Trade (Chiang Mai) Co. Ltd., Chiang Mai, Thailand. Food grade CMC (FVH6-3, DS = 0.65–0.85) was procured from Guoyu Environmental S&T Co. Ltd., Changzhou, Jiangsu, China. LNR (Mastex brand) was procured from Mastex Co. Ltd., Nakornpathom, Thailand.

### Sample preparation

CS and glycerol (CSG) were mixed at a ratio of 70/30 (% w/w) with distilled water (50 g/100 mL) through agitation in a water bath at 80 °C for 30 min. CMC was dissolved in distilled water (1 g/10 mL) at 80 °C for 10 min. LNR was incorporated into the CSG solution during the mixing process, followed by the addition of CMC solution. The concentration of CMC was added at 2–20 phr (parts/hundred) of CSG. The formulations of the CSG/LNR/CMC blends are listed in Table 2. The solutions were cast on a clean glass plate and then dried in a hot-air oven at 60 °C for 24 h.

**Table 2:** Designation and formulation of the CSG/LNR/CMC blends.

Sample	CSG (wt%)	LNR (wt%)	CMC (phr/CSG)
CSG/LNR	90	10	–
CSG/LNR/CMC2	90	10	2
CSG/LNR/CMC5	90	10	5
CSG/LNR/CMC10	90	10	10
CSG/LNR/CMC20	90	10	20

### Tensile properties

The tensile properties of the samples were evaluated in quintuplicate using a tensile tester (Tensilion UTM-II-20; Orientec Co. Ltd., Tokyo, Japan) at a crosshead speed of 2 mm/min. Bone-shaped samples were prepared using a die-cutting mold with gauge lengths, widths, and thicknesses of 10, 3, and 0.2 mm, respectively.

### Scanning electron microscopy

Morphological images of the samples were obtained using scanning electron microscopy (SEM; SM-200, Topcon Corp., Tokyo, Japan). The samples were broken in liquid nitrogen prior to the extraction of the fractured surfaces of the rubber phase using toluene at 60 °C for 1 h. The extracted fractured surfaces of the samples were coated with a thin layer of gold and measured at an acceleration voltage of 10 kV. The particle sizes of the rubber were calculated using the ImageJ software.

### Swelling measurement

The swelling percentage of the samples in water was measured for a specimen size of 50 mm × 50 mm × 0.05 mm (width × length × thickness). The samples were dried at 60 °C for 24 h and soaked in 50 mL of distilled water at 25 °C for 24 h. The swelling ratio was averaged over five samples using Eq. (1)^65^.

1 $$\mathrm{Swelling\, ratio }(\mathrm{\%})=\frac{\mathrm{Wa}-\mathrm{Wb }}{\mathrm{Wb}}\times 100$$

where W_a_ is the swollen weight and W_b_ is the dried weight.

### Solubility measurement

The sample size was 50 mm × 50 mm × 0.05 mm (width × length × thickness). The sample films were dried at 60 °C for 24 h and placed in a 250 mL Erlenmeyer flask containing 50 mL of distilled water. The samples were shaken at 25 rpm for 24 h using a shaker (OS-300, Hysc Lab, Scilution Co. Ltd., Nonthaburi, Thailand). The supernatant was filtered and the remaining samples were collected. The residue on the filter paper was dried in a hot-air oven at 80 °C for 24 h. The water solubility percentage was calculated in quintuplicate using Eq. (2)^66^.

2 $$\mathrm{Solubility }(\mathrm{\%})=\frac{\mathrm{W}1-\mathrm{W}2 }{\mathrm{W}1}\times 100$$

where W_1_ is the initial weight and W_2_ is the dried weight of the filtered sample.

### Contact angle

Drop shape analysis (DSA30E, Kruss Co. Ltd., Hamburg, Germany) was used to observe the water droplet contact angle. Samples were prepared by casting on a clean glass plate. Water was dropped onto the surface of the samples before recording the images at 3 min. Five samples were obtained for each condition.

### Fourier transform infrared spectrometer (FTIR)

FTIR (FT/IR-480 plus, Jasco Corp., Japan) was used to observe the reactions in the CSG/LNR/CMC blends. The samples were prepared as thin films using the solution-casting method. The measurement was performed from 600 cm^−1^ to 4000 cm^−1^ with a resolution of 4 cm^−1^.

### Statistical analysis

One-way ANOVA using SPSS software was used to analyze the results. Statistically significant differences at a confidence interval of 95% (*P* < 0.05) were estimated using Duncan’s test over five samples.
