# Author Correction: Corn starch reactive blending with latex from natural rubber using Na^+^ ions augmented carboxymethyl cellulose as a crosslinking agent

**DOI:** 10.1038/s41598-021-00916-0

**Published:** 2021-11-09

**Authors:** Noppol Leksawasdi, Thanongsak Chaiyaso, Pornchai Rachtanapun, Sarinthip Thanakkasaranee, Pensak Jantrawut, Warintorn Ruksiriwanich, Phisit Seesuriyachan, Yuthana Phimolsiripol, Charin Techapun, Sarana Rose Sommano, Toshiaki Ougizawa, Kittisak Jantanasakulwong

**Affiliations:** 1 School of Agro-Industry, Faculty of Agro-Industry, Chiang Mai University, Mae Hia, Muang, Chiang Mai Thailand; 2 Cluster of Agro Bio-Circular-Green Industry, Faculty of Agro-Industry, Chiang Mai University, Mae Hia, Muang, Chiang Mai Thailand; 3 Center of Excellence in Materials Science and Technology, Faculty of Science, Chiang Mai University, Mae Hia, Muang, Chiang Mai Thailand; 4 Department of Pharmaceutical Sciences, Faculty of Pharmacy, Chiang Mai University, Mae Hia, Muang, Chiang Mai Thailand; 5 Plant Bioactive Compound Laboratory (BAC), Department of Plant and Soil Sciences, Faculty of Agriculture, Chiang Mai University, Mae Hia, Muang, Chiang Mai Thailand; 6 Department of Chemistry and Materials Science, Tokyo Institute of Technology, Meguro-ku, Tokyo, Japan

Correction to: *Scientific Reports* 10.1038/s41598-021-98807-x, published online 28 September 2021

The original version of this Article contained a repeated error in Figure 1 where the code name of the rubber “LNR" was incorrectly given as “LPN”.

The original Figure 1 and accompanying legend appears below.

**Figure 1:**
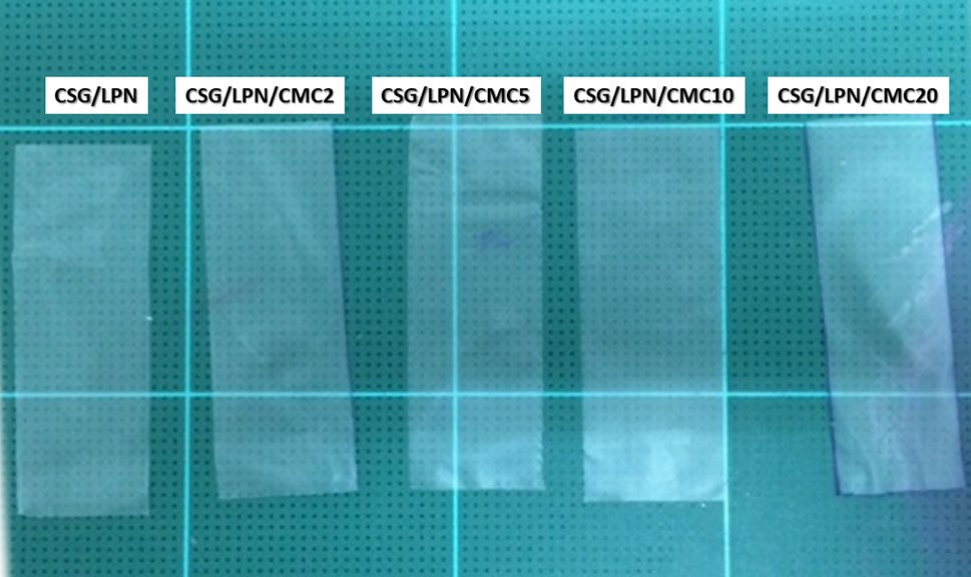
Image of CSG/LNR films blending with CMC 0, 2, 5, 10, 20 phr.

The original Article has been corrected.

